# Updated Recommendations for the Use of Typhoid Vaccine — Advisory Committee on Immunization Practices, United States, 2015

**Published:** 2015-03-27

**Authors:** Brendan R. Jackson, Shahed Iqbal, Barbara Mahon

**Affiliations:** 1 Division of Foodborne, Waterborne, and Environmental Diseases, National Center for Emerging and Zoonotic Infectious Diseases, CDC; 2 Division of Healthcare Quality Promotion, National Center for Emerging and Zoonotic Infectious Diseases, CDC

These revised recommendations of the Advisory Committee on Immunization Practices update recommendations published in *MMWR* in 1994 (1) and include updated information on the two currently available vaccines and on vaccine safety. They also include an update on the epidemiology of enteric fever in the United States, focusing on increasing drug resistance in *Salmonella enterica* serotype Typhi, the cause of typhoid fever, as well as the emergence of *Salmonella* serotype Paratyphi A, a cause of paratyphoid fever, against which typhoid vaccines offer little or no protection.

## Introduction

*Salmonella enterica* serotypes Typhi and Paratyphi A, Paratyphi B (tartrate negative), and Paratyphi C cause a protracted bacteremic illness referred to respectively as typhoid and paratyphoid fever, and collectively as enteric fever. Enteric fever can be severe and even life-threatening. It is most commonly acquired from water or food contaminated by the feces of an infected person. The incubation period is 6–30 days, and illness onset is insidious, with gradually increasing fatigue and fever. Malaise, headache, and anorexia are nearly universal. A transient macular rash can occur. When serious complications (e.g., intestinal hemorrhage or perforation) occur, it is generally after 2–3 weeks of illness. Untreated illness can last a month (2). Patients with untreated typhoid fever were reported to have case-fatality rates >10% (3); the overall case-fatality rate with early and appropriate antibiotic treatment is typically <1% (4).

*Recommendations for routine use of vaccines in children, adolescents, and adults are developed by the Advisory Committee on Immunization Practices (ACIP). ACIP is chartered as a federal advisory committee to provide expert external advice and guidance to the Director of the Centers for Disease Control and Prevention (CDC) on use of vaccines and related agents for the control of vaccine-preventable diseases in the civilian population of the United States. Recommendations for routine use of vaccines in children and adolescents are harmonized to the greatest extent possible with recommendations made by the American Academy of Pediatrics (AAP), the American Academy of Family Physicians (AAFP), and the American College of Obstetricians and Gynecologists (ACOG). Recommendations for routine use of vaccines in adults are harmonized with recommendations of AAFP, ACOG, and the American College of Physicians (ACP). ACIP recommendations approved by the CDC Director become agency guidelines on the date published in the* Morbidity and Mortality Weekly Report *(*MMWR*). Additional information is available at http://www.cdc.gov/vaccines/acip.*

Typhoid fever is uncommon in the United States, with an average of about 400 cases reported annually during 2007–2011 (5). Approximately 90% of U.S. cases occur among persons returning from foreign travel, and >75% of travelers had been in India, Bangladesh, or Pakistan (5). Most travelers (≥55%) reported that their reason for travel was visiting friends or relatives (5). Even short-term travel to high-incidence areas is associated with risk for typhoid fever (6). CDC recommends typhoid vaccination for travelers to many Asian, African, and Latin American countries, but, as of 2010, no longer recommends typhoid vaccine for travelers to certain Eastern European and Asian countries (7); the most recent pre-travel vaccination guidelines are available at http://wwwnc.cdc.gov/travel.

The importance of vaccination and other preventive measures for typhoid fever is heightened by increasing resistance of *Salmonella* serotype Typhi to antimicrobial agents, including fluoroquinolones, in many parts of the world (8).

Paratyphoid fever, caused primarily by *Salmonella enterica* serotype Paratyphi A, but also by serotypes Paratyphi B (tartrate negative) and C, is an illness clinically indistinguishable from typhoid fever (9). Serotype Paratyphi A is responsible for a growing proportion of enteric fever cases in many countries, accounting for as much as half of the cases (8). Neither typhoid vaccine available in the United States is licensed by the Food and Drug Administration for prevention of paratyphoid fever, although limited observational data suggest the oral, live-attenuated Ty21a vaccine might offer some protection against Paratyphi B (tartrate negative) (10).

## Typhoid Vaccines

Two typhoid vaccines are available for use in the United States: 1) a Vi capsular polysaccharide vaccine for parenteral use (Typhim Vi, manufactured by Sanofi Pasteur) and 2) an oral live-attenuated vaccine (Vivotif, manufactured from the Ty21a strain of *Salmonella* serotype Typhi by PaxVax). A parenteral heat-phenol-inactivated whole-cell vaccine first licensed by Wyeth in 1952 and associated with high rates of fever and systemic reactions was discontinued in 2000 (6).

No efficacy studies among travelers from nonendemic areas are available for either vaccine, though a Ty21a vaccine challenge study among North American volunteers demonstrated significant protection from disease (11,12). The two currently available vaccines have moderate efficacy in populations where typhoid is endemic. In a systematic review and meta-analysis, the estimated 2.5–3.0 year cumulative efficacy was 55% (95% confidence interval [CI] = 30%–70%) for the parenteral Vi polysaccharide vaccine and 48% (CI = 34%–58%) for the oral Ty21a vaccine, each based on a single trial (13). A trial in Kolkata, India, of the Vi polysaccharide vaccine found a protective effectiveness of 61% (CI = 41%–75%) among all participants (14). Studies conflict regarding the effectiveness of the Vi vaccine in young children. The trial in Kolkata, which included adults as well as children, found 80% (CI = 53%–91%) effectiveness among those 2–4 years (14), whereas a trial in Karachi, Pakistan, which included only children 2–16 years, showed no protection among children 2–4 years (15). Herd effects might have contributed to the high effectiveness observed among young children in the Kolkata trial. An observational study of the effectiveness of typhoid vaccination in U.S. travelers estimated 80% protection; however, this study addressed typhoid vaccination in general, not specific vaccines (16).

Protein-conjugated Vi polysaccharide vaccines have been shown to have high efficacy in young children (17) and have been licensed in other countries (18), but are not currently licensed or available in the United States.

## Vaccine Usage

Routine typhoid vaccination is not recommended in the United States.

Vaccination is recommended for the following groups:

- Travelers to areas where there is a recognized risk for exposure to *Salmonella* serotype Typhi (the most recent guidelines are available at http://wwwnc.cdc.gov/travel). Risk is greatest for travelers who have prolonged exposure to possibly contaminated foods and beverages, although short-term travelers are also at risk (6). Most travel-associated typhoid fever cases in the United States occur among travelers who are visiting friends or relatives; many travelers in this group do not seek pre-travel health care (19). Multidrug-resistant strains of *Salmonella* serotype Typhi have become common in many regions (8), and cases of typhoid fever that are treated with drugs to which the organism is resistant can be fatal. Travelers should be cautioned that typhoid vaccination is not a substitute for careful selection of food and beverages. Typhoid vaccines are not 100% effective, and vaccine-induced protection can be overwhelmed by large inocula of *Salmonella* serotype Typhi.
- Persons with intimate exposure (e.g., household contact) to a documented *Salmonella* serotype Typhi chronic carrier (defined as excretion of *Salmonella* serotype Typhi in urine or stool for >1 year).
- Microbiologists and other laboratory workers routinely exposed to cultures of *Salmonella* serotype Typhi or specimens containing this organism or who work in laboratory environments where these cultures or specimens are routinely handled.

## Choice of Vaccine

Parenteral Vi polysaccharide and oral Ty21a are both acceptable forms of typhoid vaccine.

The Vi polysaccharide vaccine is administered as a single injection and is approved for adults and children aged ≥2 years. The oral Ty21a vaccine is administered in 4 doses on alternating days over 1 week and is approved for adults and children aged ≥6 years. Immunocompromised persons should not use Ty21a because it is a live-attenuated vaccine. Because antibacterial drugs might be active against the vaccine strain and reduce immunogenicity, the Ty21a vaccine should not be administered to persons taking these medications.

## Vaccine Administration

### Vi polysaccharide

Primary vaccination with Vi polysaccharide consists of one 0.5-mL (25-*μ*g) dose administered intramuscularly. This vaccine should be given at least 2 weeks before potential exposure.

### Ty21a

Primary vaccination with live-attenuated Ty21a vaccine consists of one enteric-coated capsule taken on alternate days (day 0, 2, 4, and 6), for a total of four capsules. The capsules must be kept refrigerated (not frozen). Each capsule should be taken with cool water no warmer than 98.6°F (37.0°C), approximately 1 hour before a meal. All doses should be completed at least 1 week before potential exposure.

### Repeat Doses

If continued or repeated exposure to *Salmonella* serotype Typhi is expected, repeat doses of typhoid vaccine are needed to maintain immunity (Table). An optimal revaccination schedule for the Vi polysaccharide vaccine has not been established; however, the manufacturer recommends a repeat dose every 2 years after the primary dose if continued or renewed exposure is expected (20). The manufacturer of Ty21a recommends revaccination with the entire 4-dose series every 5 years if continued or renewed exposure to *Salmonella* serotype Typhi is expected (21).

## Adverse Reactions

Evidence from trials and postmarketing studies suggest that parenteral Vi vaccines are usually tolerated well (20). In field trials, pain (risk ratio [RR] = 8.0; CI = 3.7–17.2) and swelling at the injection site (RR = 6.0; CI = 1.1–34.2) were more common among vaccinees than placebo recipients, but no significant difference was found in the incidence of fever or erythema (13). In a manufacturer-funded postmarketing safety study conducted in 11 U.S. travel clinics, the most common reactions were injection site pain (77%), tenderness (75%), and muscle aches (39%) (22). In postmarketing surveillance of the Vi vaccine (administered alone or simultaneously with other vaccines) during 1995–2002, an estimated 0.3 serious events* per 100,000 doses distributed were reported to the U.S. Vaccine Adverse Events Reporting System (VAERS) (23). Among the 321 VAERS reports of events occurring after Vi vaccination, the most commonly reported symptoms included injection site reactions, fever, headache, rash, urticaria, abdominal pain, and nausea. It is important to note that adverse events reported to VAERS might not be caused by the vaccine.

In a meta-analysis of Ty21a vaccine placebo-controlled trials, fever was more common among vaccinees (RR = 1.8; CI = 1.0–3.1), but other adverse events occurred with equal frequency among groups receiving vaccine and placebo; risk for any mild adverse event was higher among vaccinees (RR = 1.7; CI = 1.0–2.7) (13). In a combined analysis of data from a pilot study and a field trial, fewer than 10% of vaccinees reported abdominal pain (6.4%), nausea (5.8%), headache (4.8%), fever (3.3%), diarrhea (2.9%), vomiting (1.5%), or skin rash (1.0%) (21,24,25). One nonfatal case of anaphylactic shock, which was considered to be an allergic reaction to the vaccine, was reported to the manufacturer (21). In VAERS postmarketing surveillance of the Ty21a vaccine (administered alone or simultaneously with other vaccines) during 1991–2002, an estimated 0.6 serious events per 100,000 doses distributed were reported (23). Among the 345 reports of events occurring after Ty21a vaccination, the most commonly reported symptoms included diarrhea, nausea, fever, abdominal pain, headache, rash, vomiting, and urticaria (23).

## Precautions and Contraindications

No data have been reported on the use of either typhoid vaccine in pregnant women. In general, live vaccines like Ty21a are contraindicated in pregnancy (26). Vi polysaccharide vaccine should be given to pregnant women only if clearly needed (20).

Because Ty21a is a live-attenuated vaccine, antimicrobial agents might interfere with vaccine activity. To be sure the vaccine is fully effective, the vaccine manufacturer advises that Ty21a should not be given until at least 3 days after the last dose of antimicrobial agent and, if possible, antimicrobial agents should not be started within 3 days of the last dose of Ty21a vaccine (27). A longer interval should be considered for long-acting antimicrobials (e.g., azithromycin). The antimalarial agents mefloquine and chloroquine and the combinations atovaquone/proguanil and pyrimethamine/sulfadoxine can, at doses used for prophylaxis, be administered together with the Ty21a vaccine; however, the manufacturer advises that other antimalarial agents only be administered at least 3 days after the last vaccine dose (27). Ty21a vaccine can be administered simultaneously or at any interval before or after other live vaccines (injectable or intranasal) or immune globulin if indicated (26). Ty21a should not be administered to persons during an acute febrile illness or acute gastroenteritis (21).

Live-attenuated Ty21a vaccine should not be used by immunocompromised persons. The Vi vaccine is theoretically safer for this group. Although the Ty21a strain can be shed in the stool of vaccinees, transmission has not been documented (21). The Ty21a strain has not been isolated from blood cultures after vaccination (21). Both the Vi polysaccharide and Ty21a vaccines are contraindicated in patients with a history of hypersensitivity to any component of the vaccine.

**What is currently recommended?**

In 1994, Advisory Committee on Immunization Practices (ACIP) approved recommendations for typhoid vaccination, stating that typhoid vaccine is indicated for U.S. travelers to certain countries, close contacts of chronic carriers, and certain laboratory workers. Since 1994, the parenteral heat-phenol-inactivated whole-cell vaccine has been discontinued.

**Why are the recommendations being modified now?**

The updated recommendations contain new data on the epidemiology of typhoid fever and vaccine effectiveness and safety. No substantive changes have been made to ACIP typhoid vaccine recommendations apart from removing the discontinued parenteral whole-cell vaccine from the list of available typhoid vaccines. The two typhoid vaccines available in the United States are parenteral Vi capsular polysaccharide vaccine and oral live-attenuated Ty21a vaccine.

**What are the new recommendations?**

Typhoid vaccine continues to be recommended for U.S. travelers to certain countries (the most recent guidelines are available at http://wwwnc.cdc.gov/travel), close contacts of chronic carriers, and certain laboratory workers.

## Figures and Tables

**TABLE:** Updated dosage and schedules for typhoid fever vaccination — Advisory Committee on Immunization Practices, United States, 2015

		Dosage			
		
Vaccination	Age (yrs)	Dose/mode of administration	No. of doses	Dosing schedule	Boosting interval
**Vi capsular polysaccharide vaccine**					
Primary series	≥2	0.50 mL*	1	1 dose	—
Booster	≥2	0.50 mL*	1	1 dose	Every 2 yrs
**Oral live-attenuated Ty21a vaccine**					
Primary series	≥6	1 capsule†	4	Days 0, 2, 4, 6	—
Booster	≥6	1 capsule†	4	Days 0, 2, 4, 6	Every 5 yrs

* Intramuscularly.

† Each orally administered capsule contains 2.0–10.0 × 10^9^ viable *Salmonella enterica* serotype Typhi Ty21a and 5–50 × 10^9^ nonviable *Salmonella enterica* serotype Typhi Ty21a.

## Footnotes

* Serious adverse events were defined as reports of death, hospitalizations, prolongation of hospitalization, permanent disability, life-threatening illness, or congenital anomaly.
